# Psychosocial Functioning, BMI, and Nutritional Behaviors in Women at Cardiovascular Risk

**DOI:** 10.3389/fpsyg.2020.02135

**Published:** 2020-08-28

**Authors:** Khaya N. Eisenberg, Elisheva Leiter, Rivka T. May, Tanya Reinfeld, Donna R. Zwas

**Affiliations:** Linda Joy Pollin Cardiovascular Wellness Center for Women, Division of Cardiology, Hadassah University Medical Center, Jerusalem, Israel

**Keywords:** cardiovascular risk, health behaviors, anxiety, depression, stress, emotional eating, physical activity

## Abstract

Psychosocial factors such as depression, anxiety, and stress are associated with increased cardiovascular risk. Health behaviors may play a role in this relationship, as individuals experiencing elevated levels of anxiety, stress, and/or depression may be less likely to engage in risk-reducing behaviors such as diet and exercise. Some evidence suggests that this relationship is particularly relevant for women. This study explored the relationship between levels of anxiety, depression, stress, and specific nutritional behaviors in a sample of 187 women at cardiovascular risk. BMI was explored as a possible moderator of these relationships. Higher levels of depression in patients with high BMI was associated with increased fruit consumption, whereas this was not seen in highly depressed patients with normal BMI. The reverse pattern was seen for consumption of sweet drinks. Anxiety was found to have a complex relationship with consumption of sweetened drinks and white bread, with higher consumption at moderately elevated levels of anxiety and reduced consumption at the highest levels. Possible interpretations of these findings, as well as their implications for lifestyle interventions with this population are discussed. These findings suggest a number of questions for further research.

## Introduction

Psychosocial factors such as depression, stress, and anxiety are associated with increased cardiovascular risk (Fan et al., 2008). People who suffer depression are at greater risk for cardiovascular disease, and patients with comorbid cardiovascular disease and depression have a higher mortality rate. The risk increases for those with more severe depression (Hare et al., 2014). Psychological stress is also described in the 2016 European clinical guidelines for the prevention of cardiovascular disease as a factor that may contribute to both the development and progression of cardiovascular disease (Piepoli et al., 2016). Although the evidence for the impact of depression and stress is more consistent, multiple studies suggest that chronic anxiety may also have a deleterious effect on cardiovascular health (Cohen et al., 2015). According to one meta-analysis (Roest et al., 2010), anxiety is associated with a 26% increase in risk of incident coronary heart disease as well as a 48% increase in risk of cardiac death.

The mechanisms by which psychological factors influence cardiovascular illness are not fully understood, but health behaviors may play a role (Cohen et al., 2015). Some researchers have suggested that individuals at cardiovascular risk with comorbid psychiatric disorders may adopt behaviors that run counter to risk reduction (Szlejf et al., 2019). The relationship between psychological distress and negative health behaviors is well-supported. For example, one study found an association between general emotional distress and poorer exercise behaviors, i.e., greater sedentariness and reduced physical activity (St.-Pierre et al., 2019). Psychological distress has also been found to predict greater consumption of palatable, non-nutritious foods (e.g., Groesz et al., 2012). Psychological factors may augment the challenges faced by individuals at cardiovascular risk who seek to adopt positive health behaviors (Rippe and Angelopoulos, 2014). Addressing these factors, therefore, could significantly impact the effectiveness of lifestyle interventions.

The relationship between emotional factors and health behaviors for individuals at cardiovascular risk is particularly relevant for women. Rates of stress and depression are higher in women (Vaccarino and Bremner, 2017), and both the presence and severity of anxiety disorders are higher in women than in men (Serpytis et al., 2018). Compared with men, women have been found to be more disturbed by psychosocial stress (Beutel et al., 2018). Additionally, the effects of anxiety on cardiovascular health vary by gender. For example, the presence of an anxiety disorder was found to be associated with higher odds of compromised cardiovascular health among women, but not in men (Szlejf et al., 2019).

Psychological functioning appears to have a differential effect on health behaviors for men and for women. One study of cardiac patients found that women with cardiovascular disease and comorbid depression were less likely to engage in physical activity compared with their male counterparts (Allabadi et al., 2019). In one particularly striking study, higher levels of anxiety for women were associated with a significant decrease in a score measuring healthy lifestyle (i.e., physical activity, diet, BMI, and alcohol and tobacco consumption) over 20 years. This effect was independent of measured healthy lifestyle at the onset of the study; women with high anxiety had reduced odds of maintaining a healthy lifestyle over 20 years even if their lifestyle had been healthy initially (Trudel-Fitzgerald et al., 2016).

This study, focusing on a sample of women at high risk for cardiovascular disease, explored the relationship between psychological functioning and health behaviors. Our goal was to understand the particular impact of depression, anxiety, and stress on specific nutrition behaviors in this population. We were also interested in whether this relationship would vary for patients with high BMI as opposed to normal BMI. Emotional eating has been found to be associated with high BMI (Lazarevich et al., 2016); as such, we speculated that the relationship between psychosocial factors and unhealthy eating choices would be stronger for those with a high BMI than for those with a low BMI. This study builds on previous studies which examined psychological stress as a global construct (e.g., St.-Pierre et al., 2019) or evaluated healthy lifestyle as a whole (e.g., Trudel-Fitzgerald et al., 2016), taking a closer look at the relationship between individual emotional factors and specific eating behaviors.

## Materials and Methods

### Study Sample

This sample included 187 consecutive patients seen at the Linda Joy Pollin Cardiovascular Wellness Center for Women (LJPCWW) at Hadassah University Medical Center between January 2014 and August 2019. LJPCWW is a prevention clinic aimed at reducing cardiovascular mortality in women. Patients may be referred by their doctor or self-referred. Patients were included in this study if they had undergone a cardiovascular event (myocardial infarction, percutaneous coronary intervention, or stroke), had an active cardiac symptom (e.g., chest pain or arrhythmia), or had three or more active risk factors (i.e., diabetes, hypertension, hyperlipidemia, peripheral artery disease, current smoker, family history of premature coronary disease, gestational diabetes, pregnancy-induced hypertension/pre-eclampsia, or obesity). Patients were excluded if they were pregnant, had type 1 diabetes, a psychiatric diagnosis that precluded participation, dementia, or if they were under the care of another multi-disciplinary clinic.

### Data Collection

Data was collected using self-report questionnaires completed by the patients at their initial clinic evaluation.

### Measures

Demographic data included age, education, and monthly income. Patients’ weight and height were measured by the nurse, and their BMIs were calculated using the standard BMI equation (weight divided by height-squared).

Depression, anxiety and stress were measured utilizing a validated translation of the DASS-21 (Depression, Anxiety, and Stress Scale) (Lovibond and Lovibond, 1995/2019). The DASS-21 is a valid and reliable self-report scale assessing depression, anxiety and stress, which has been widely used in clinical and non-clinical populations. The DASS-21 is a set of three subscales of seven items each: depression, anxiety, and stress. Subjects are asked to use four-point severity/frequency scales to rate the extent to which they have experienced each state “over the past week.” Scores for Depression, Anxiety and Stress are calculated by summing the scores for the relevant items; higher scores are consistent with more severe symptomatology. Normal, mild, moderate, and severe depression, anxiety, and stress were determined based on the scoring system published for the scale.

Nutrition behaviors were measured using questions from a culturally adapted translation of the Healthy Heart Score (Chiuve et al., 2014) which is based on questions from the food frequency questionnaire of the Nurses Health Study (2019) (available at https://www.nurseshealthstudy.org/participants/questionnaires; accessed 27-11-19). Self-reported data was collected from patients with regard to consumption of fruits and vegetables, whole grains, white flour, sugar-sweetened beverages, and red and processed meats. For each food item, participants were asked how often, on average, a specified portion was consumed during the past week. In accordance with the Healthy Heart Score, consumption of fruits, vegetables, and whole grains were considered health-promoting nutrition behaviors. Consumption of white flour, sugar-sweetened beverages, and red and processed meats were considered non-health promoting nutrition behaviors.

### Statistical Analysis

Demographic and other quantitative data were analyzed with SPSS version 25 (IBM SPSS Statistics, Armonk, NY, United States).

These data were collected from community participants as part of an existing research program; as such, the number of respondents was fixed. As such, sample power was calculated a posteriori for a multiple-regression analysis with four independent variables and effect size = 0.05 (Faul et al., 2009). This *post hoc* power analysis (*N* = 180; significance level = 0.05; effect size = 0.05; k_measurement_ = 4) yielded a power (1-β) of 0.85.

Descriptive statistics and regression analyses were used. Linear and curvilinear regressions were used to assess the effects of the psychological predictors (i.e., anxiety, depression, and stress) together with BMI as a physiological predictor. All dependent variables were continuous, although some were measured on an ordinal (Likert) scale or count. In each linear model engagement in positive and negative nutrition behaviors (i.e., consumption of health-promoting foods or non-health-promoting foods) were measured as a function of the two predictors, psychological and physiological.

To test for potential confounding effects, we correlated three possible confounders with the measures of depression, anxiety, and stress. We also correlated these factors with BMI, which we expected to affect the daily consumption of various food products. None of the background variables were shown to have potential confounding effects. Since these variables did not show any correlation with the independent measures, any potential confounding effect would be insignificant notwithstanding their association with the daily consumption of various food products.

Two additional tests were added to this model: (1) A curvilinear association between the measured psychological variables and eating habits; and (2) An interaction effect between BMI and the psychological predictors. The first test was meant to assess the possibility of a non-linear association between psychological factors and eating behaviors, i.e., that the association between psychological factors and eating behaviors would change direction after a certain point. The second was a test of non-independency between the two indicators, i.e., for varying BMI levels, the association between the psychological indicators and eating behaviors would change, and vice versa. We presented interaction results in the high-low approach (Aiken and West, 1991) and the point estimation approach (Johnson and Neyman, 1936). Results are presented in tables and relevant charts.

### Ethical Considerations

Human subject approval was obtained from the Hadassah Medical Organization Institutional Review Board, and the study conformed to the principles outlined in the Declaration of Helsinki (Study # HMO- 0419-17).

## Results

Demographic data describing the participants are presented in Table 1, and descriptive statistics in Table 2. The average age of patient participants was 59.3 (*SD* = 11.12), with a range of 25–95. 37% of the participants were overweight (BMI = 25–29.9), and 40% were obese (BMI > 29.9). Mean depression (3.81), anxiety (3.83), and stress (6.20) levels were higher than would be expected in the general population. No differences were seen in the prevalence of depression, anxiety or stress in diabetic, hypertensive or hyperlipidemic patients (*F* = 0.247–2.11; *p* = 0.147–0.619). No differences in the prevalence of depression, anxiety, or stress were found for any of the demographic variables.

**TABLE 1:** Demographic data.

**Characteristic**	**Percentage**
Age	
<40	5.0
41–50	14.4
51–60	26.5
61–70	41.4
71–80	11.0
>81	1.7
Marital status	
Married	75.8
Separated/divorced	10.2
Widowed	9.7
Single	4.3
Level of education	
Elementary School	3.3
High School Equivalency	16.5
High School Diploma	17.6
Professional Certificate	3.8
Bachelor’s Degree	37.4
Graduate Degree	21.4
Monthly income	
Up to 4000 NIS	12.9
4001–7500 NIS	33.1
7001–15000 NIS	47.2
Over 15000 NIS	6.7

**TABLE 2:** Descriptive statistics.

**Variable**	**Categories**	**Count**	**Percent**	**Means**	***SD***
					
Age		181		59.93	11.12
BMI		179		29.20	5.71
	Normal	43	23		
	Overweight	64	36.9		
	Obese	72	40.2		
Depression				3.81	3.92
	Normal	100	70.4		
	Mild-moderate	29	20.4		
	Severe-extremely severe	13	9.2		
	Total	142	100		
Anxiety				3.83	3.73
	Normal	83	57.2		
	Mild-moderate	38	26.2		
	Severe-extremely severe	24	16.6		
	Total	145	100		
Stress				6.20	4.47
	Normal	93	65.0		
	Mild-moderate	34	23.8		
	Severe-extremely severe	16	11.2		
	Total	143	100		
Hyperlipidemia	Yes	100	54.6		
Hypertension	Yes	55	30.1		
Systolic		180		130.41	18.71
Diastolic		178		70.78	9.88
Diabetes	Yes	29	16.1		

### Depression, BMI, and Nutrition Behaviors

Regression analyses of the relationship between depression and BMI and eating behaviors are presented in Table 3.

**TABLE 3:** Regression analyses for depression, BMI, and food consumption, standardized coefficients.

	**Fruit per day**	**Vegetable per day**	**Whole grains**	**Sweet drinks per day**	**White bread per day**	**Red meat per day**
Step 1						
BMI	0.08	−0.02	−0.02	0.19*	0.20*	−0.15
Dep.	0.16	−0.01	0.11	0.03	0.17	−0.05
*R*^2^	0.03	0.01	0.01	0.03	0.06*	0.03
Step 2a						
Dep.	−0.10	−0.44*	0.15	0.13	0.31	0.18
Dep.^2^	0.30	0.39*	−0.05	−0.12	−0.16	−0.26
Δ*R*^2^	0.02	0.04*	0.00	0.00	0.01	0.02
*R*^2^	0.05	0.05	0.01	0.04	0.07*	0.04
Step 2b						
BMI X Dep.	0.19*	0.02	0.18	−0.19*	−0.03	−0.06
Δ*R*^2^	0.03*	0.00	0.03	0.03*	0.001	0.004
*R*^2^	0.06*	0.01	0.05	0.07*	0.06	0.03

*p < 0.05; Step 1, linear; Step 2a, curvilinear; Step 2b, interaction; Dep, depression.

**FIGURE 1:**
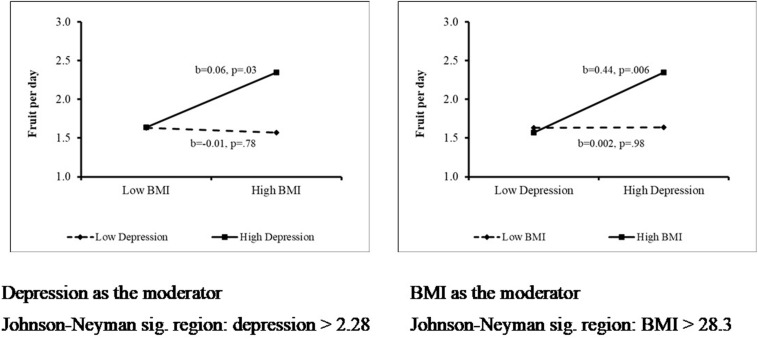
Interaction effect of BMI and depression on daily fruit consumption.

When the effects of depression and BMI on daily fruit consumption were analyzed using linear regression, results were not significant. However, assessment of interaction effects revealed that for individuals with higher levels of depression, BMI showed a positive association with fruit consumption (*b* = 0.06, *p* = 0.03). For individuals with lower levels of depression, BMI was not significantly associated with fruit consumption (cutoff point: depression greater than 2.28, *p* < 0.05). Additionally, for individuals with high BMI, depression was positively associated with daily fruit consumption (*b* = 0.44; *p* = 0.006). For individuals with lower BMI, this association was not significant (see Figure 1).

**FIGURE 2:**
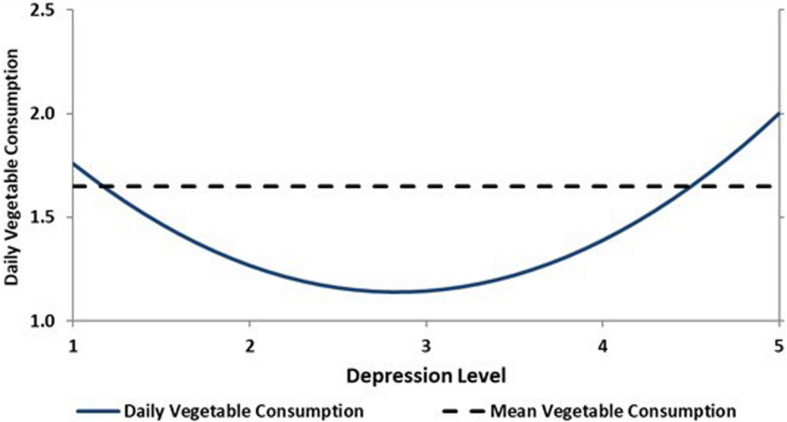
Curvilinear prediction of daily vegetable consumption by depression.

Daily vegetable consumption did not have a significant linear association with psychological variables or with BMI. However, when we tested for curvilinear effects, the association between depression and daily vegetable consumption was found to have a parabolic shape (β_linear_ = −0.44, *p* < 0.05; β_quadratic_ = 0.39, *p* < 0.05). That is, depression and daily vegetable consumption were negatively associated when depression scores were low, from normal to mild. When depression scores were higher than 1.18 (i.e., ranging from mild to extremely severe; maximum = 14+), increasing levels of depression were associated with increased vegetable consumption (see Figure 2). Note that this relationship was significant with respect to the curvilinear model. However, this model did not explain a significant percentage of the overall variance.

With regard to sweet drink consumption, higher BMI was associated with increased consumption of sweet drinks (β = 0.19, *p* < 0.05). Depression had no direct linear effects on sweet drink consumption, but the additional interaction term (model 2b) indicated differing effects of depression on sweet drink consumption in high vs. low BMI study participants. When depression was low, a positive association between BMI and sweet drink consumption was maintained (*b* = 0.04, *p* = 0.004). However, this relationship did not hold at higher levels of depression.

**FIGURE 3:**
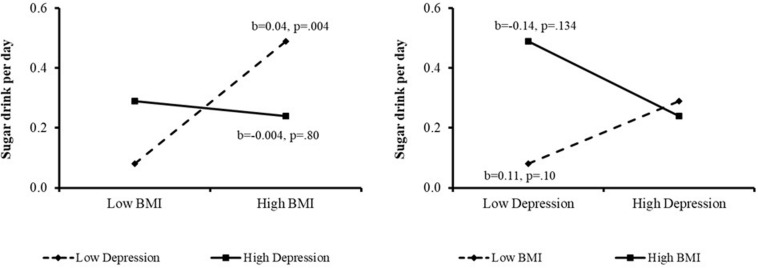
Interaction effect of BMI and depression on daily sugar drink consumption.

When we further explored this relationship, we found that in low BMI participants, consumption of sweet drinks increased as levels of depression increased. In high BMI participants, in contrast, sweet drink consumption decreased as depression increased. Both of these associations were insignificant, however, other than at the extremes of the sample. [The Johnson-Neyman analysis (see Figure 3) showed that for those whose BMI fell below 20.15, slightly below the sample mean, the association between depression and sweet drink consumption was positive, while this association was negative for those whose BMI exceeded 49, two standard deviations above the sample mean].

In addition, we found a linear positive association between BMI and daily consumption of white bread (β = 0.20, *p* < 0.05). Consumption of whole grains or red and processed meats was not affected by depression or by BMI, neither alone nor in interaction.

### Anxiety, BMI, and Nutrition Behaviors

We examined the effects of anxiety and BMI on nutrition (see Table 4). Anxiety was not found to significantly affect daily consumption of fruit or daily consumption of vegetables, alone or in interaction with BMI. No linear relationship was found between anxiety and consumption of sweet drinks, alone or in interaction with BMI.

**TABLE 4:** Regression results for anxiety, BMI, and food consumption, standardized coefficients.

	**Fruit per day**	**Vegetable per day**	**Whole grain**	**Sweet drinks per day**	**White bread per day**	**Red meat per day**
Step 1						
BMI	0.06	0.03	−0.02	0.15	0.13	−0.14
Anx.	0.05	−0.04	−0.02	0.16	0.21*	−0.02
*R*^2^	0.01	0.00	0.00	0.06*	0.07*	0.02
Step 2a						
Anx.	0.08	0.00	−0.09	0.65*	0.53*	0.23
Anx.^2^	−0.04	−0.05	−0.08	−0.61*	−0.40*	−0.31*
Δ*R*^2^	0.00	0.00	0.00	0.13*	0.06*	0.03*
*R*^2^	0.01	0.00	0.00	0.19*	0.13*	0.05
Step 2b						
BMI X Anx.	−0.05	−0.07	−0.02	0.03	−0.02	0.01
Δ*R*^2^	0.002	0.01	0.001	0.001	0.00	0.00
*R*^2^	0.01	0.01	0.001	0.06	0.07*	0.02

*p < 0.05; Step 1, linear; Step 2a, curvilinear; Step 2b, interaction; Anx, anxiety.

**FIGURE 4:**
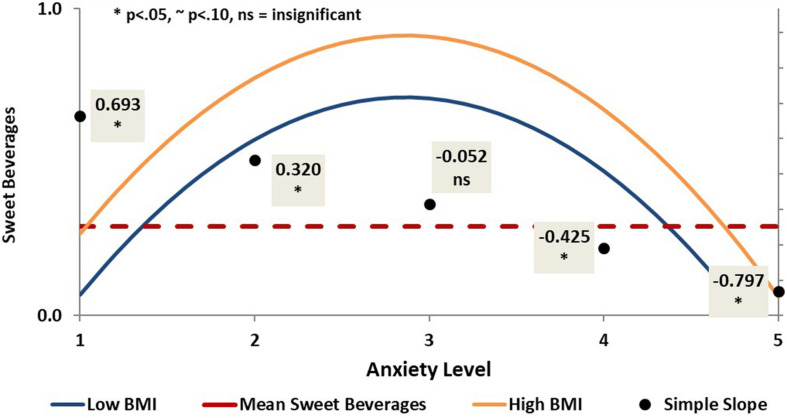
Curvilinear prediction of daily sweetened drink consumption by anxiety.

However, anxiety did have a significant curvilinear relationship (β_linear_ = −0.65, *p* < 0.05; β_quadratic_ = −0.61, *p* < 0.05) with consumption of sweetened drinks (see Figure 4). Specifically, at low and mild levels of anxiety, as anxiety increased, consumption of sweet drinks increased. The direction of this relationship reversed for patients with severe and extremely severe levels of anxiety, such that as anxiety increased, daily consumption of sweet drinks decreased. The direction of this relationship was not affected by BMI.

**FIGURE 5:**
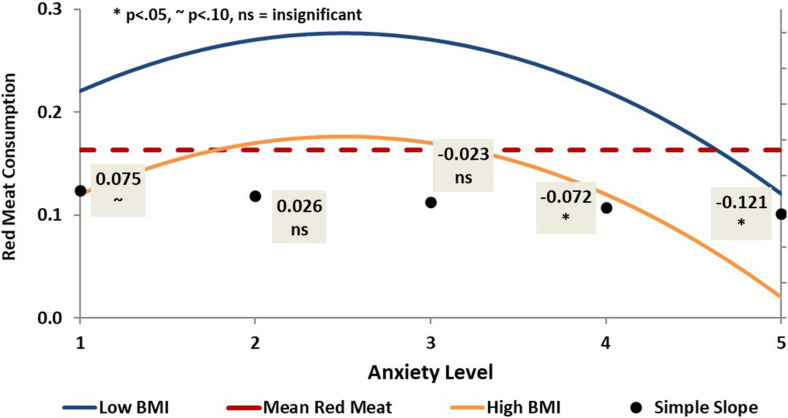
Curvilinear prediction of red and processed meat consumption by anxiety.

Anxiety was also found to have a curvilinear relationship (β_linear_ = 0.23, ns; β_quadratic_ = −0.31, *p* < 0.05) with consumption of red and processed meats (see Figure 5), although this relationship was only significant at severe and extremely severe levels of anxiety. Specifically, red and processed meat consumption showed a slight (non-significant) increase as anxiety increased for patients with normal and mild levels of anxiety. After anxiety reached a moderate level, consumption of red and processed meat decreased as anxiety increased to severe and extremely severe levels.

**FIGURE 6:**
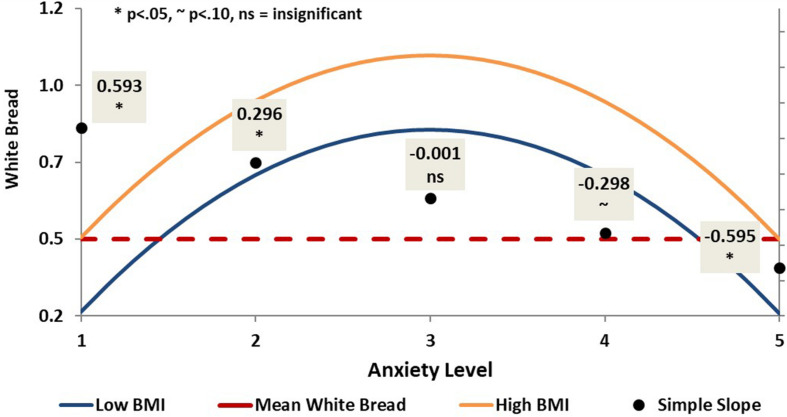
Curvilinear prediction of white bread consumption by anxiety.

Finally, anxiety displayed a significant curvilinear relationship (β_linear_ = 0.53, *p* < 0.05; β_quadratic_ = 0.40, *p* < 0.05) with consumption of white bread (see Figure 6). For patients with normal and mild levels of anxiety, increased anxiety led to increased consumption of white bread (*p* < 0.05). For patients with moderate and severe levels of anxiety, this relationship appeared to reverse its direction with increased anxiety correlating with decreased consumption of white bread, although the relationship was no longer significant at these levels. As anxiety increased to extremely severe levels, the effects of anxiety on white bread consumption reached significance but were now negative, with increased anxiety leading to decreased white bread consumption. Anxiety did not have a significant effect on consumption of whole grains, neither alone nor in interaction with BMI.

### Stress, BMI, and Nutritional Behaviors

We finally examined the effects of stress and BMI on eating behaviors (see Table 5). Although BMI was consistently found to predict daily sweetened drink consumption and daily white bread consumption, this relationship was not affected by changes in stress levels. Level of stress was not found to affect any of the nutrition behaviors studied. No significant linear, curvilinear, or interaction effects were found for stress and fruit and vegetable consumption, stress and sweet drink consumption, stress and red and processed meat consumption, stress and white bread consumption, or stress and whole grain consumption.

**TABLE 5:** Regression results for stress, BMI, daily activity and diet consumption, standardized coefficients.

	**Fruit per day**	**Vegetable per day**	**Whole grain**	**Sweet drinks per day**	**White bread per day**	**Red meat per day**
Step 1						
BMI	0.06	−0.01	−0.03	0.19*	0.19*	−0.14
Stress	0.02	−0.03	0.11	0.09	0.12	0.13
*R*^2^	0.00	0.00	0.01	0.04	0.05	0.04
Step 2a						
Stress	0.33	−0.12	0.19	0.37	0.39	0.35
Stress^2^	−0.33	0.10	−0.09	−0.30	−0.29	−0.23
Δ*R*^2^	0.01	0.00	0.00	0.01	0.01	0.01
*R*^2^	0.02	0.00	0.01	0.05	0.06	0.05
Step 2b						
BMI X Stress	0.02	−0.13	0.12	−0.18	−0.02	−0.16
Δ*R*^2^	0.00	0.01	0.01	0.02	0.00	0.01
*R*^2^	0.004	0.01	0.02	0.06	0.05	0.05

*p < 0.05; Step 1, linear; Step 2a, curvilinear; Step 2b, interaction.

## Discussion

This research examined the relationship between reported levels anxiety, depression, and stress and nutrition behaviors, i.e., specific food consumption practices in a sample of women at cardiovascular risk. It also examined whether having a high BMI would affect these relationships. We focused specifically on health-promoting consumption (i.e., fruit, vegetable, and whole grain consumption) and non-health promoting consumption (i.e., sweetened drink, white bread, and red and processed meat consumption). With regard to health-promoting food consumption, we found that depression was related to consumption of fruits and vegetables. With regard to non-health promoting food consumption, we found that anxiety was related to consumption of sweetened drinks, white bread, and red and processed meats.

### Depression and Consumption of Health-Promoting Foods

With regard to health-promoting food consumption, we examined the relationship between BMI, psychological factors, and fruit and vegetable intake. Our study found no relationship between fruit and vegetable consumption and level of anxiety or stress, regardless of BMI level, but did find an association between depression and fruit and vegetable consumption, moderated by BMI in the case of fruit consumption. For high-BMI individuals, increased depression was associated with greater intake of fruits. The relationship between depression and fruit consumption was not significant for low-BMI individuals. One meta-analysis found that increased fruit and vegetable consumption were associated with a lower risk of depression (Liu et al., 2016) although the relationship between depression and fruit and vegetable consumption has not been consistently supported (e.g., Whitaker et al., 2014; Wu et al., 2018). Other studies have found that increased fruit and vegetable consumption predicted were associated with reduced depression and also reduced anxiety (e.g., McMartin et al., 2013).

This result is somewhat surprising in light of previous findings that fruit consumption was negatively associated with obesity (e.g., Porter Starr et al., 2014) as well as with depression (Liu et al., 2016). It is worth noting that it was difficult to find studies exploring the interaction of BMI and depression with regard to fruit consumption. Our findings may suggest that, notwithstanding the fact that high-BMI individuals may be generally less likely to eat fruit, high-BMI individuals with depression may actually consume more fruit. Conversely, although depressed individuals may generally consume less fruit, depressed individuals with high BMI may consume more fruit. More research is needed to understand the particular combined impact of BMI and depression with regard to fruit consumption.

The relationship between depression and vegetable consumption in our study was also multifaceted although in this case, BMI did not influence this relationship. As depression increased from normal to mild levels, vegetable consumption decreased. This tendency then reversed itself, with vegetable consumption increasing as depression increased from mild to severe and extremely severe levels.

In light of other studies that found that increased depression was generally associated with lower consumption of vegetables (e.g., Payne et al., 2012), our finding that this was only the case for patients with normal-to-mild levels of depression is quite surprising. It was even more surprising to find that the reverse was true for patients with more severe depression. Although the overall model for this analysis was not significant and the implications of this finding are therefore more tentative, it is worth exploring whether the relationship between depression and vegetable consumption might change for a subgroup of highly depressed patients. Obtaining a further understanding of the complex relationship between depression and fruit and vegetable consumption is especially important since fruit and vegetable consumption may partially mediate the relationship between depression and progression of heart disease (Rutledge et al., 2014).

Our research found no association between whole grains and BMI, depression, anxiety, or stress. This contrasts with previous research suggesting that whole grain consumption is associated with decreased anxiety (Sadeghi et al., 2017) as well as lower depressive symptoms (Rius-Ottenheim et al., 2017). On the other hand, other studies found no association for depression and whole grain consumption (e.g., Grases et al., 2019) or for whole grain consumption and reduced distress (e.g., Soltani et al., 2018). If there is a relationship between psychological functioning and consumption of whole grains, these conflicting results suggest that other factors may need to be examined for their impact on this relationship.

### Depression, Anxiety, and Non-health Promoting Food Consumption

In exploring the relationship between psychological factors, BMI, and consumption of non-health promoting food items, we examined daily consumption of red and processed meats as well as simple carbohydrates, i.e., sweetened drinks and white bread. With regard to red and processed meats, in our sample consumption demonstrated a curvilinear relationship with anxiety was albeit only significant at severe and extremely severe levels of anxiety. At severe and extremely severe levels of anxiety, red and processed meat consumption were seen to decline as anxiety increased. In contrast, other research has found a positive association between anxiety and a diet high in both sugars and saturated fat (e.g., Masana et al., 2019) or specifically high in saturated fat from meat and processed foods in particular (e.g., Yannakoulia et al., 2008; see Dutheil et al., 2016). Our finding may be a function of our unique population, an older group of Israeli women. While red and processed meat has become more popular in Israel, this is a relatively recent change (Endevelt et al., 2017). It is possible that our sample of older Israeli women may eat less red and processed meat in general, and that the relationship between anxiety and consumption of red and processed meat for these women may not be generalizable to the broader population.

With regard to consumption of simple carbohydrates, it was not surprising to find that high BMI was associated with both greater consumption of sweetened drinks and greater consumption of white bread. This is consistent with research indicating that consumption of sugar-sweetened beverages is correlated with obesity risk (for a recent review, see Luger et al., 2017), as is white bread consumption (e.g., de la Fuente-Arrillaga et al., 2014). In our population, however, the relationship between BMI and daily sweetened drink consumption was moderated by level of depression.

Specifically, for patients at low levels of depression, daily sweet drink consumption increased as BMI increased. For patients with more severe depression, sweetened drink consumption did not increase with increased BMI. Our findings may suggest that, notwithstanding the fact that in general high-BMI individuals may be more likely to consume sweetened drinks, this may not be the case for high-BMI individuals who also have depression.

This is consistent with the fact that our research also found no direct relationship between depression and sweet drink consumption. This contrasts with previous research which identified an association between depression and sweetened beverages (e.g., Knüppel et al., 2017; Huang et al., 2019). Some of these findings on this relationship were a bit more ambiguous on closer examination, though. For example, in one study this association was more relevant for diet drinks (Guo et al., 2014) and did not seem to be related to the drink’s sugar content *per se*. Another study suggesting a preference for sweetened foods among women with depressive symptoms reported small effect sizes (Jeffery et al., 2009). Clearly, the association of increased depression with increased sweetened drink consumption is not unequivocal.

Our findings reflect the possibility that elevated levels of depression include anhedonia and reduced motivation to seek sensory pleasure. The relevance of this to sweetened drinks in particular is illustrated by one study of rats bred to be highly susceptible to developing learned helplessness (a precursor to depression). These rats were found to be less motivated to press levers in order to drink a sugar solution (Vollmayr et al., 2004). Increased depression, then, might not lead to a desire for sweets and increased sweet drink consumption.

Anxiety, on the other hand, was found to have a complex relationship with daily sweetened drink consumption. We found that, irrespective of BMI level, at normal and mild levels of anxiety, increased anxiety predicted a higher number of sweetened drinks consumed per week. In contrast, more severe levels of anxiety predicted fewer sweetened drinks consumed per week. This tendency was similar with regard to consuming white bread. At lower levels of anxiety, irrespective of BMI, increased anxiety predicted an increase in servings of white bread consumed per week, whereas more severe levels of anxiety predicted fewer servings of white bread consumed per week.

That this study found a significant relationship between sweet drink consumption and anxiety is not surprising. Research demonstrates that many individuals tend to eat tasty, non-nutritious foods in response to distress (Groesz et al., 2012). Concerning sweetened foods, both the metabolic and pleasurable properties of sugar are believed to reduce the intensity of the stress response, suggesting that some people may overindulge in sugary foods as a means of relieving stress (Ulrich-Lai, 2016). With regard to sweet drinks in particular, an earlier study found that consumption of a sugar-sweetened drink, in contrast to a drink that was artificially sweetened, increased calmness for participants who had experienced an acute stressor (Samant et al., 2016).

While a tendency to consume sweet drinks may be a form of self-medication for individuals with anxiety, the relationship between sugar consumption and anxiety might be bidirectional. The Western diet, based on a high consumption of added sugar as well as other high calorie low nutrient foods (Casas et al., 2018), is associated with anxiety (Jacka et al., 2011). One study of rats found that a diet high in refined carbohydrates increased vulnerability to stress (Santos et al., 2018). These findings could suggest a vicious cycle whereby increased anxiety and increased consumption of sweet drinks are mutually self-perpetuating.

The relationship between anxiety and white bread consumption may be similarly bidirectional. According to some research, long-term consumption of sugar results in reduced basal dopamine levels, which may trigger the desire to overindulge in carbohydrate-rich foods in order to return dopamine to homeostatic levels (Jacques et al., 2019). As with sweet drinks, if consuming foods high in refined flour is an attempt to self-medicate, it may be misguided. Although some authors have suggested an association between carbohydrate-rich foods and improved mood (e.g., Christensen and Pettijohn, 2001), more recent research counters this (Matantzis et al., 2019). It is possible that a craving for carbohydrate-rich foods and negative mood are mutually reinforcing, perpetuating a cycle of anxiety and unhealthy eating.

### Anxiety and Consumption of Simple Carbohydrates: Proposed Physiological Explanations

As noted earlier, a physiological mechanism may underlie the relationship between anxiety and difficulty resisting food cravings. Much research on anxiety has suggested that increased anxiety is associated with autonomic nervous system dysfunction, particularly reduced parasympathetic nervous system activity and reduced heart rate variability (see Sperry et al., 2018 for a brief review), although these findings are not uniform. While some research has resulted in similar findings for depression, these findings have been more heterogeneous and might be more accurately attributed to comorbid anxiety rather than to the specific impact of depression (Rottenberg, 2007).

Adaptive levels of heart rate variability, a measure of autonomic nervous system functionality, are associated with flexible emotional responding to the environment and self-regulation (Thayer et al., 2009). Self-regulation includes the ability to modulate one’s response to food cravings. Not surprisingly, heart rate variability is positively associated with the ability to regulate one’s eating behavior (Meule et al., 2012a,b). In fact, higher levels of sympathetic activation, as measured by decreased heart rate variability, may be associated with increased levels of craving and decreased self-regulation (Quintana et al., 2013; Moretta and Buodo, 2018). Taken together, this suggests that as anxiety increases and heart rate variability decreases, one would expect to see a corresponding decrease in self-regulation and in the ability to resist unhealthy food cravings. Since the relationship between depression and heart rate variability has been proposed to be a function of comorbid anxiety (Rottenberg, 2007), it makes sense that giving in to unhealthy food cravings would be more predictable for patients with anxiety than for patients with depression.

These findings, though, would likely predict a direct linear relationship between increased anxiety and simple carbohydrate consumption. As such, the curvilinear relationship between anxiety and simple carbohydrate consumption found in this study is surprising. Our findings show that while respondents who reported lower levels of anxiety were most prone to consuming simple carbohydrates as anxiety increased, respondents who reported severe anxiety were far less likely to indulge. This would suggest that simple carbohydrate consumption increases together with greater anxiety up to a particular threshold, and then decreases.

Polyvagal theory may lie at the root of our curvilinear findings. According to polyvagal theory (Porges, 2009), the autonomic nervous system has evolved over time while retaining vestiges of older systems. The newest and most sophisticated responses to stress involve a range of behavior and physiological states. The fight-or-flight response is an earlier, more primitive reaction to an environmental challenge. And the earliest and most primitive reaction to a threat is passive avoidance, or immobilization.

Perhaps the curvilinear relationship we found between anxiety and non-health promoting eating behaviors mirrors neurophysiological regression to increasingly primitive systems. As anxiety increases from normal to mild and moderate levels, the physiological response may revert from more adaptive responses to more primitive fight-or-flight responses, involving an increase in cortisol (see Porges, 2009) which has been implicated in increased sweet food consumption (Epel et al., 2001). But as anxiety increases from moderate to severe levels, the body’s response may switch from the increased cravings associated with cortisol and fight-or-flight, to the more primitive passive avoidance or immobilization response. The latter response may predict a withdrawal from comfort eating and other means of self-soothing.

The direction of our curvilinear findings is surprising, though, if we take a closer look at the relationship between anxiety, autonomic functioning, and self-regulation. As noted above, increased anxiety appears to be associated with decreased heart rate variability and deficits in self-regulation. For example, anxiety has been conceptualized as a compromised ability to respond flexibly to the environment, particularly with regard to continuing to feel threatened despite the absence of a verified threat (Thayer and Lane, 2000). This self-regulatory ability has been proposed to be directly associated with cardiac vagal tone, i.e., the vagus nerve’s ability to regulate the heart rate (as measured through heart rate variability) (Kogan et al., 2013).

Interestingly, the relationship between cardiac vagal tone and well-being has also been suggested to be curvilinear. Patients with low-to-moderate cardiac vagal tone were found to have a positive linear link between cardiac vagal tone and well-being. That is, as cardiac vagal tone increased, well-being showed a corresponding increase. However, for patients in the moderate-to-high range of cardiac vagal tone, increased cardiac vagal tone was found to have no relationship, or even a negative relationship, with well-being (Kogan et al., 2013).

If cardiac vagal tone is a direct index of anxiety and self-regulation, than individuals with lower vagal tone should have higher anxiety and poorer self-regulation. For these high-anxiety individuals, as vagal tone improves and anxiety decreases, there should be a corresponding improvement in self-management, including the ability to regulate one’s eating. Yet we found that at higher levels of anxiety, reduced anxiety was actually associated with increased consumption of refined carbohydrates. In our study, the tendency to succumb to unhealthy food cravings appeared to be greater for those with moderate anxiety than for those with high anxiety.

One possible explanation for this finding is that the physiological response underlying anxiety may not be as well-understood as is commonly believed. In fact, some researchers have suggested that although anxiety is frequently assumed to have a predictable underlying physiological response based on self-reported physical symptoms (e.g., rapid heartbeat, sweating, and hyperventilation), when physiological indicators of anxiety are measured objectively, their presence and intensity are not always consistent with patient self-report. In fact, some findings have suggested that patients with higher, more chronic anxiety have reduced, rather than intensified, physiological responses in contrast to their perceived experience (see Lang and McTeague, 2009).

Notwithstanding the widely accepted view that anxiety is uniformly associated with autonomic reactivity and reduced heart rate variability, several researchers suggest that anxiety disorders are physiologically heterogeneous. Autonomic responses to psychological stresses have actually been found to vary from individual to individual (Berntson and Cacioppo, 2004) as well as from anxiety disorder to anxiety disorder. For example, in contrast to patients with more situationally triggered anxiety (e.g., simple phobias), patients with more diffuse chronic anxiety have been found in some studies to actually to have lower levels of measured physiological responsiveness. Defensive physiological responses may be blunted for individuals with high, chronic anxiety (Lang and McTeague, 2009). Additional research has supported the conclusion that the more enduring, wide-ranging, and intense the negative affect across a variety of anxiety disorders, the greater the reduction in physiological reactivity (McTeague and Lang, 2012). Respondents whose DASS-21 anxiety scores fell at the severe end likely fall into this category. This may offer a physiological explanation for our findings that at high levels of anxiety, emotional eating appeared to decrease rather than continuing to increase in the expected direction.

### Anxiety and Consumption of Simple Carbohydrates: Proposed Psychological Explanations

From a psychological perspective, it is possible that while the temptation to overindulge in sweet drinks may increase together with anxiety for those who are less anxious, those experiencing more extreme levels of anxiety may feel too overwhelmed to attempt to reduce their tension through this particular means of self-regulation. As with sweet drink consumption, the curvilinear relationship between anxiety and white bread consumption, which starts out positive and then becomes negative as anxiety increases to severe levels, may be a function of learned helplessness and an inability to self-soothe through emotional eating at extreme levels of anxiety. It is possible that as individuals become more than moderately emotional dysregulated, other coping mechanisms – more dangerous ones, perhaps – take the place of emotional eating.

Comfort eating as an attempt to self-soothe may be most relevant for those whose anxiety has reached a moderate level, whereby they are feeling sufficiently taxed so as to engage in negative health behaviors as a means of self-soothing and not too paralyzed to do so. This is actually a reversal of the Yerkes–Dodson inverted U-shaped performance law (Yerkes and Dodson, 1908), which suggests that performance is enhanced by an optimal amount of arousal and compromised at lower and higher amounts.

An application of the Yerkes-Dodson Law to the relationship between psychosocial functioning and health behaviors might predict that individuals at cardiovascular risk reporting moderate levels of anxiety would be most likely to engage in health-promoting behaviors while individuals who report normal or severe levels would be less committed to adhering to health behaviors. Yet, findings of this study displayed the opposite pattern.

Of note, in rodent models, acute and chronic distress have been associated with decreased food intake and weight loss, and increased caloric efficiency (Rabasa and Dickson, 2016). Human studies have demonstrated that distress is associated with increased preference for palatable food and central weight gain (Wardle et al., 2000). It is possible that animal stress models represent a high stress situation, compared to more moderate stressors in the human study.

With regard to anxiety in particular, another possibility might be that individuals with high anxiety are more prone to social desirability bias and, as a result, likely to be less accurate when reporting their health behaviors. Studies suggest that individuals asked to report on their risky behaviors may not always be consistent or accurate in their reporting (e.g., Palen et al., 2008). People with high social desirability bias may be uncomfortable providing accurate information on their health behaviors when these behaviors are contrary to common medical and public health advice (Latkin et al., 2017).

In relation to self-reported nutrition behaviors, several studies have found that social desirability bias can influence underreporting of calorie intake, particularly in women (for a review, see Maurer et al., 2006). In fact, one study of 450 post-menopausal women, a population resembling that of the current study, found that participants who scored high in social desirability were more likely to underreport their calorie intake (Mossavar-Rahmani et al., 2013). Some research suggests a possible connection between elevations in traits such as neuroticism or social anxiety and inauthentic self-presentation (e.g., Twomey and O’ Reilly, 2017). Taken together, it is plausible that as anxiety increased in this sample, a tendency to underreport negative health behaviors may have increased as well, resulting in a decrease in reported soft drink consumption for respondents with severe or very severe anxiety.

### Lack of Findings for Stress

It is interesting to note that, in this study, we found no relationship between stress as measured by the DASS-21 and level of physical activity, positive eating habits, or negative eating habits. This might be a function of the DASS-21 scoring system, where thresholds for increased stress scores are higher than those for mild and moderate depression and anxiety scores, requiring a greater frequency and degree of endorsed stress-related items to earn a higher score. Additionally, studies suggest that while the DASS-21 showed high correlations with established measures with regard to depression and anxiety, stress as measured by the DASS-21 has more heterogeneous associations with psychological distress (Alfonsson et al., 2017). As such, measurement fluctuations may have contributed to this result.

A physiological explanation is also possible. Some researchers have suggested that whereas anxiety may be associated with high levels of autonomic activation, stress may be associated with reduced cardiovascular activation (Borkovec and Hu, 1990). Others have argued that no single pattern of autonomic activity and heart rate variability will manifest uniformly across a variety of stresses, given that the concept of stress is vague and poorly defined in the literature. Studies of the autonomic response to psychological stress found a great deal of variability between individuals (Berntson and Cacioppo, 2004). While our findings suggest that the physiological reaction to anxiety may be more heterogeneous and complex than the literature would suggest, the available research indicates that the physiological reaction to stress might be even less predictable and consistent than the physiological response to anxiety.

### Limitations and Strengths

This study is limited by a moderate rather than large sample size, which may lead to lowered sensitivity to subtle behavioral differences. The *post hoc* power analysis, however, suggested that the sample size was sufficient for the performance of these analyses.

Another limitation of this study was its use of self-report with regard to diet, which calls accuracy into question. Social desirability bias, unreliable memory, and other factors could affect precise reporting of eating habits. In particular, anxiety and depression may affect recall (Hertel and Brozovich, 2010) which may have ramifications for the nutrition behaviors reported by participants with greater anxiety and depression. The differences found between high BMI and low BMI participants with depression, however, suggests that depression itself did not account for the differences in reported behaviors. Similarly, the curvilinear responses to anxiety suggests that this is not a linear response to anxiety.

With regard to psychological functioning, depression, anxiety, and stress were measured with a brief questionnaire which could not fully assess all of the psychological symptoms a respondent may have been experiencing at the time of the study. We chose this measure because it has been extensively used to identify psychological factors in cardiovascular patients and individuals at risk for CVD (e.g., Nekouei et al., 2014; Kang et al., 2019) and has been validated in Hebrew. The measure does not, however, provide a complete picture of psychological functioning.

Finally, this study only examined women at risk of heart disease who voluntarily presented at a preventive health clinic. The results may not be generalizable to men. Additionally, these women’s choosing to seek out preventive heart care suggests a level of motivation and attention to their health which may not be characteristic of a more general population. The characteristics of our population may be unique in other ways as well. Almost 60% of our respondents had completed college, with more than one-third of this subgroup having completed graduate degrees. This study may also reflect cultural factors associated with the local population, including the high prevalence of adherence to the Mediterranean diet. Results from this population may not be generalizable to populations with more heterogeneity with regard to education or nutrition culture.

This study also had a number of strengths. Participants’ weight and height were assessed objectively, as opposed to using self-report for all measures. Additionally, data collection was conducted at patients’ initial evaluation, before an intervention might have impacted on participants’ nutritional habits. Finally, the inclusion of a broad spectrum of highly anxious and depressed individuals as well as a broad range of BMIs in our study contributed to the robustness of our conclusions.

### Implications and Future Research

Our findings showed that, for this population of women at cardiovascular risk, increasing anxiety at lower levels of anxiety are related to increased consumption of sugars and refined grains, while this relationship changed direction at more severe levels of anxiety. Severe anxiety was also correlated with a decrease in processed meat consumption. These findings were consistent for both normal BMI and high BMI patients. These curvilinear relationships between psychosocial functioning and nutrition behaviors may suggest that unhealthy eating behaviors are most affected from mild to moderate levels of psychopathology. For depressed patients, BMI was associated with different nutrition behaviors at lower and higher levels of depression.

Anxiety is often assumed to have a predictable association with autonomic nervous system activity, which has also been implicated in the physiology of food cravings and self-regulation of eating behavior. Our findings suggest that the physiological reaction to anxiety, in general and as it relates to food cravings and self-regulation, may be more multifaceted than is often supposed. The physiology behind anxiety and food cravings is an important area of study, especially for researchers interested in cardiovascular health. Heart rate variability and vagal tone, which are implicated in anxiety, self-regulation, and eating behavior, have significant implications for cardiac wellness. A better understanding of this complex relationship could lead to multi-level interventions that could simultaneously address anxiety, nutrition, and cardiovascular health.

Future research could examine the relationship between particular levels of anxiety and particular ways of self-soothing in a population at cardiovascular risk. In particular, qualitative research using clinical interviews might focus on eliciting specific ways that individuals in this population attempt to regulate their emotions at varying levels of psychosocial functioning.

These findings have implications for those designing lifestyle interventions to decrease cardiovascular risk. In attempting to help individuals at cardiovascular risk, it is clearly important not only to screen for anxiety, but to assess the level at which these qualities may be present. Emotional eating may be most relevant, or most helpful to address, for those with mild to moderate levels of anxiety. Different levels of anxiety may have different effects on health behaviors and other means of coping. These are questions which future studies should explore.

Lifestyle interventions aimed at decreasing cardiovascular risk often focus on improving diet and exercise. However, weight loss with these interventions is often not maintained (Wu et al., 2009). Even interventions specifically focused on maintaining weight loss show modest and heterogeneous effects (Dombrowski et al., 2014). Our study points to the importance of evaluating and treating psychological functioning as well as individual eating behaviors as part of a lifestyle intervention.

To address the needs of women at cardiovascular risk who present with mild-to-moderate levels of anxiety, interventions specifically targeting emotional eating behaviors should address the underlying mechanisms contributing to maintaining weight loss after dieting. Specifically, mindfulness-based interventions appear to be a promising approach (O’Reilly et al., 2014). Mindfulness practices, particularly those which emphasize the impermanence of experiences, have been found to reduce impulsive and high-calorie eating as well as perceived cravings (Keesman et al., 2017). Mindfulness-based approaches could have the dual benefit of addressing eating behavior and reducing depression, anxiety, and stress (see Janssen et al., 2018).

Our findings support the need to identify and treat mental health issues in women at cardiovascular risk when implementing lifestyle interventions. Our findings also suggest that the level at which these issues are present is particularly relevant. In particular, moderate levels of anxiety appear to be most predictive of engaging in unhealthy comfort eating behaviors. Women at cardiovascular risk presenting with these levels of anxiety would likely benefit most from interventions providing alternatives to emotional eating and teaching positive coping skills, whereas women with higher levels of anxiety may benefit from treatment of anxiety prior to initiation of lifestyle interventions. Women with higher levels of depression and with high BMI may benefit from a tailored approach that differs from depressed women with low BMI.

## Data Availability Statement

The datasets generated for this study are available on request to the corresponding author.

## Ethics Statement

The studies involving human participants were reviewed and approved by Hadassah Medical Organization Institutional Review Board. The patients/participants provided their written informed consent to participate in this study.

## Author Contributions

KE contributed to analysis or interpretation of data for the work, drafting the manuscript, and critical revision for important intellectual content. EL and DZ contributed to the conception and design of the work, the acquisition, analysis or interpretation of data, drafting the manuscript, and critical revision for important intellectual content. RM contributed to analysis and interpretation of data for the work, drafting the manuscript, and critical revision for important intellectual content. TR assisted with acquisition of data. All authors approved the publication of this content and agreed to be accountable for all aspects of the work in ensuring that questions related to the accuracy or integrity of any part of the work are appropriately investigated and resolved.

## Conflict of Interest

The authors declare that the research was conducted in the absence of any commercial or financial relationships that could be construed as a potential conflict of interest.
